# The Health Impact of Rabies in Haiti and Recent Developments on the Path Toward Elimination, 2010–2015

**DOI:** 10.4269/ajtmh.16-0647

**Published:** 2017-10-18

**Authors:** Ryan Wallace, Melissa Etheart, Fleurinord Ludder, Pierre Augustin, Natael Fenelon, Richard Franka, Kelly Crowdis, Patrick Dely, Paul Adrien, J. Pierre-Louis, Modupe Osinubi, Lillian Orciari, Marco Vigilato, Jesse Blanton, Roopal Patel, David Lowrance, Andrecy Liverdieu, Andre Coetzer, John Boone, Joanne Lindenmayer, M. Millien

**Affiliations:** 1 U.S. Centers for Disease Control and Prevention, Poxvirus and Rabies Branch, Atlanta, Georgia;; 2 U.S. Centers for Disease Control and Prevention, Port-au-Prince, Haiti;; 3 Department of Animal Health, Ministry of Agriculture, Natural Resources, and Rural Development, Port-au-Prince, Haiti;; 4 Pan American Health Organization, Port-au-Prince, Haiti;; 5 Christian Veterinary Mission, Port-au-Prince, Haiti;; 6 Directorate of Epidemiology, Laboratory, and Research, Ministry of Public Health and Population, Port-au-Prince, Haiti;; 7 Directorate of Public Sanitation and Public Education, Ministry of Public Health and Population, Haiti;; 8 Global Alliance for Rabies Control, Pretoria, South Africa;; 9 Department of Microbiology and Plant Pathology, Faculty of Natural and Agricultural Sciences, University of Pretoria, South Africa;; 10 Humane Society International, Washington, District of Columbia

## Abstract

Haiti, a Caribbean country of 10.5 million people, is estimated to have the highest burden of canine-mediated human rabies deaths in the Western Hemisphere, and one of the highest rates of human rabies deaths in the world. Haiti is also the poorest country in the Western Hemisphere and has numerous economic and health priorities that compete for rabies-control resources. As a result, primary rabies-control actions, including canine vaccination programs, surveillance systems for human and animal rabies, and appropriate postbite treatment, have not been fully implemented at a national scale. After the 2010 earthquake that further hindered the development of public health program infrastructure and services, the U.S. Centers for Disease Control and Prevention worked with the Ministry of Public Health and Population and key health development partners (including the Pan-American Health Organization) to provide technical expertise and funding for general disease surveillance systems, laboratory capacity, and selected disease control programs; including rabies. In 2011, a cross-ministerial rabies consortium was convened with participation from multiple international rabies experts to develop a strategy for successful rabies control in Haiti. The consortium focused on seven pillars: 1) enhancement of laboratory diagnostic capacity, 2) development of comprehensive animal surveillance system, 3) development of comprehensive human rabies surveillance system, 4) educational outreach, 5) sustainable human rabies biologics supply, 6) achievement of sustained canine vaccination rates of ≥ 70%, and 7) finalization of a national rabies control strategy. From 2010 until 2015, Haiti has seen improvements in the program infrastructure for canine rabies control. The greatest improvements were seen in the area of animal rabies surveillance, in support of which an internationally recognized rabies laboratory was developed thereby leading to an 18-fold increase in the detection of rabid animals. Canine rabies vaccination practices also improved, from a 2010 level of approximately 12% to a 2015 dog population coverage level estimated to be 45%. Rabies vaccine coverage is still below the goal of 70%, however, the positive trend is encouraging. Gaps exist in the capacity to conduct national surveillance for human rabies cases and access to human rabies vaccine is lacking in many parts of the country. However, control has improved over the past 5 years as a result of the efforts of Haiti’s health and agriculture sectors with assistance from multiple international organizations. Haiti is well situated to eliminate canine-mediated human rabies deaths in the near future and should serve as a great example to many developing countries struggling with similar barriers and limitations.

## INTRODUCTION

Rabies is responsible for an estimated 59,000 deaths globally each year, more than any other zoonotic disease in the world.^1,2^ Dogs are the most significant reservoirs for rabies virus in terms of public health and pose the greatest risk to people.^3^ Primary rabies interventions, therefore, focus on control of the disease in affected dog populations through vaccination, population management, and responsible ownership practice.^4^ Secondary interventions to prevent human deaths rely on pre-exposure (PrEP), which is rarely applied in developing countries, or post-exposure prophylaxis (PEP), which is not sustainable for most governments if implemented without control of rabies in animals.^5^ Today, the canine rabies virus can be found in more than 150 countries, placing approximately half of the world’s human population at risk of becoming exposed.^3^

Rabies is one of the oldest recorded zoonotic diseases, with passages describing this disease first appearing in written literature as early as 2,000 bc in the Middle East.^6^ For centuries, canine-mediated human rabies deaths were primarily a threat in Europe and Asia. However, with colonial expansion in the fifteenth and sixteenth centuries and the relatively long incubation period of rabies in dogs, European colonists brought both dogs and the disease with them to the new world. France first colonized the Caribbean island of Hispaniola in 1659, and with them came rabies infected dogs. The presence of animal rabies on the island was first reported by French veterinarian Jean Lompardieu LAPOLE in a thesis entitled: *Observations on the Health of the Animals of St Domingue 1788*. There is little data on human and animal rabies cases from the eighteenth through twentieth centuries in Haiti. The next reports note that between 1970 and 1986, Haiti recorded 998 rabies cases among dogs and cats (59 per year) and in 2013–2014 recorded 101 rabid animals (50 per year).^7,8^

In many Latin American countries, through primary intervention methods implemented in the 1970s, dog-mediated human rabies deaths decreased from 350 per year to less than 10 from 1980 to 2010.^9^ Unfortunately, this success was not mirrored in Haiti, where between 1980 and 1986, 18 human rabies deaths were reported (2.6 per year).^7^ Reported rabies human deaths seem to have only increased over time as a passive human rabies surveillance system in Haiti currently detects approximately 7–17 human rabies deaths each year.^10^ Despite these counts being highest among all Western Hemisphere countries, it is largely recognized as a significant underrepresentation of the true burden as there is no laboratory-based surveillance for human rabies and medical provider awareness for this disease is low.^11^ Modeled estimates suggest this number may be in excess of 130 human rabies deaths annually.^2^

## COLLABORATING FOR RABIES CONTROL IN HAITI

Although endemicity of rabies among the local dog populations in Haiti has been known since 1788, the absence of existing infrastructure for sample collection and effective laboratory-based surveillance has precluded any comprehensive efforts to reliably quantify rabies burden. The lack of understanding of true disease burden has made it difficult to mobilize resources for the implementation of rabies control measures within local dog and human populations.^11^ Limited reports of clinically diagnosed human rabies cases (7–17 annually) and scarce instances of laboratory confirmation of dog rabies (≤ 4 cases annually) have clouded the true disease burden. However, the notable number of exported cases provide a glimpse into the underappreciated seriousness of the situation; five human rabies deaths diagnosed in Europe, United States, and Canada are attributed to infection in Haiti.^10,12–14^

In 2010, following the devastating earthquake that significantly compromised Haiti’s already vulnerable public health system and overall infrastructure, resources for rabies prevention became even scarcer. As a response to the overall crisis, the U.S. government has committed significant resources toward relief, recovery, and reconstruction efforts.^15,16^ The U.S. Centers for Disease Control and Prevention (CDC) partnered with the Haitian Ministry of Public Health and Population (MSPP), Ministry of Agriculture, Natural Resources, and Rural Development (MARNDR), and National Water and Sanitation Agency to deliver public health services, strengthen disease surveillance, and develop effective prevention and intervention programs. As a part of this large endeavor, approximately $600,000 in U.S. government funding was provided to support rabies control and prevention efforts in the country for a 5-year period (2011–2015).

In 2011, the CDC Poxvirus and Rabies Branch (PRB) initiated rabies control efforts by working with local partners to establish a rabies prevention and control consortium consisting of representatives from MSPP, MARNDR, Pan American Health Organization (PAHO), and CDC as well as nongovernmental organizations (Global Alliance for Rabies Control [GARC], Christian Veterinary Mission [CVM], and later on also Humane Society International [HSI]). This consortium conducted a gap analysis and developed a comprehensive 5-year strategy to improve rabies control capacity, based on the components of Rabies Blueprint for Canine Rabies Elimination (http://caninerabiesblueprint.org).^4^ The plan focused on seven major pillars: 1) enhancement of laboratory diagnostic capacity, 2) development of comprehensive animal surveillance system, 3) development of comprehensive human rabies surveillance system, 4) educational outreach, 5) sustainable human rabies biologics supply, 6) achievement of sustained canine vaccination rates of ≥ 70%, and 7) finalize a national rabies control strategy (Table 1).

**Table 1:** Summary of rabies control progress from 2010 to 2015 by categories developed by the Haiti rabies consortium

5-year goals	Program status: 2010	Program status: 2015
a) Enhance laboratory diagnostics	• Antiquated animal rabies diagnostic techniques	• Gold standard diagnostic methods established at Central Veterinary Laboratory
	• 0 animals tested for rabies	• 70 animals tested for rabies annually
	• No international validation of test results	• Successfully passed PAHO proficiency testing
	• No human rabies diagnostic capacity	• No human samples tested, although capacity for postmortem testing exists at Central Veterinary laboratory
b) Develop animal rabies surveillance system	• No trained animal rabies control workforce	• 40 animal rabies control officers trained; 16 hired full time by MARNDR
	• No standard case definitions	• An animal rabies surveillance system, supported by MARNDR, is implemented in three departments
	• No formal protocols for animal investigation, observation, testing, or reporting	• 1,180 rabies suspect animal investigations
	• 0 animal rabies investigations	• 75 rabid animals detected in three departments (laboratory and clinical case definitions)
	• 0 rabid animals detected, nationally	
c) Develop human rabies surveillance system	• No standard case definitions	• Case definitions developed and disseminated to sentinel hospitals
	• No standard procedures for investigation, testing, or reporting	• Standard investigation procedures under review by MSPP
	• 1 human death reported	• 7 human deaths reported in 2015
d) Develop and expand educational outreach	• Few rabies educational outreach materials	• 55,738 children received rabies prevention education in 2015
	• No standard educational materials for public health professionals	• GARC Rabies Educator Certificate course conducted; 43 professionals trained
e) Establish sustainable access to human rabies vaccine	• 20,000 human rabies vaccines donated	• 20,000 human rabies vaccines donated
	• Disseminated to only 16 of more than 1,100 health centers	• Disseminated to only 16 of more than 1,100 health centers
	• No standard bite treatment or PEP triage protocols	• Bite treatment and PEP protocols under review by PAHO, CDC, and MSPP
f) Expand canine rabies vaccination coverage to ≥ 70%	• 0 dogs vaccinated (0% vaccination coverage)	• 457,448 dogs vaccinated (45.7% estimated vaccination coverage)
	• 3-year average: 7.3% coverage	• 3-year average: 24.2%
g) Draft and finalize a national rabies control strategy	• 2007–2011 national plan drafted but not enacted	• 2016–2020 national plan under review
	• No national plan from 2011–2015	• Reviewed by MSPP, MARNDR, PAHO, and CDC
National rabies program evaluation*	• 0.0 out of 5.0	• 1.5 out of 5.0

CDC = U.S. Centers for Disease Control and Prevention; GARC = Global Alliance for Rabies Control; MARNDR = Ministry of Agriculture, Natural Resources, and Rural Development; MSPP = Ministry of Public Health and Population; PAHO = Pan American Health Organization; PEP = postexposure prophylaxis.

* Stepwise Approach towards Rabies Elimination.

## LABORATORY DIAGNOSIS

**Figure 1.**
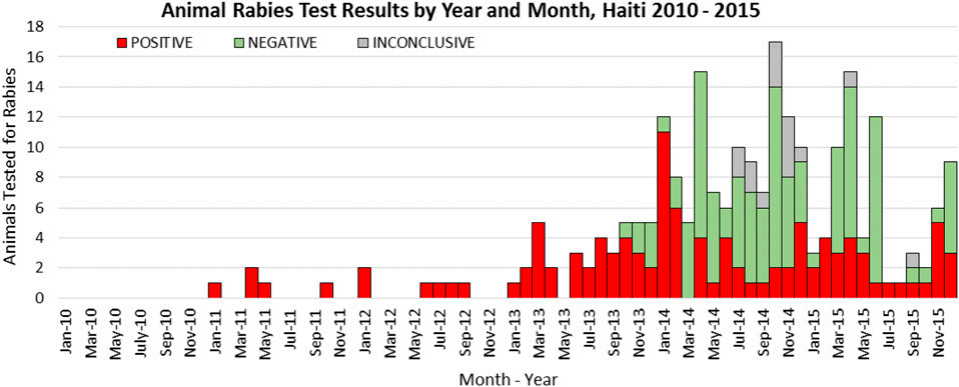
Animal rabies test results by year and month, Haiti 2010–2015.

Prior to 2011, only limited laboratory diagnostics for rabies was performed in the Central Veterinary Laboratory in Haiti. The laboratory lacked infrastructure for sample collection, transportation, and proper sample storage. Furthermore, the laboratory used the outdated Seller’s stain technique, which has shown low sensitivity for rabies virus detection compared with the gold standard Direct Fluorescent Antigen (DFA) test.^17^ As a result of earlier limitations, only very few samples (*N* = 11) were received for testing during the 3-year period 2010–2012; most samples were from the capital city of Port-au-Prince (Figure 1).

In 2011 and 2012, CDC assisted in the provision of essential laboratory equipment and reagents as well as multiple extensive hands-on trainings in proper sample collection and diagnostic testing (DFA test, as well as direct rapid immunohistochemistry test). Laboratory development and trainings were validated through twice-annual CDC confirmatory testing as well as the blind PAHO proficiency tests for Central and South America. Parallel confirmatory testing of all positive and 10% of negative samples conducted by CDC rabies laboratory since 2013 has shown 100% consistency between laboratory results among adequate samples (data not published).

Decentralization of rabies diagnostics has proven difficult given the scarcity of adequate laboratory facilities. To expand surveillance coverage in the absence of regional laboratories, CDC and MARNDR developed zoonotic disease processing stations (ZDP), facilities that met a minimum standard of biosafety to allow safe collection and temporary storage of samples from rabies suspected animals. There are now two operational ZDPs (in Center and Artibonite Departments) and one being developed for use in 2016 (in Nord Department). Since operationalizing the national laboratory and regional ZDPs, and in combination with a rabies surveillance program, the laboratory tested 37 animals in 2013, 118 in 2014, and 70 in 2015. This represents a 20-fold increase in diagnostic testing compared with the 3 years prior to implementation of the laboratory (Figure 1).

## DEVELOPMENT OF AN INTEGRATED BITE CASE MANAGEMENT SYSTEM

Primary rabies control is achieved through mass vaccination of dogs; however, in Haiti and many developing countries, it has proven difficult to reach the level of vaccination coverage required to achieve elimination (≥ 70%).^11,18^ Although Haiti’s canine rabies vaccination program continues to expand, secondary control measures were instituted in the form of an integrated bite case management (IBCM) program, modeled on programs conducted in the United States and Philippines.^19,20^ Haiti’s IBCM is a system in which the public health and agricultural sectors collaborate to investigate rabies suspect dogs involved in human exposure events. Under IBCM programs, rabies exposures (i.e., bites) are reported to a trained animal control workforce who then assess the offending animal for signs of rabies and subsequently quarantine or submit the animal for rabies testing. These results are reported to the victims as well as the public health sector in timely fashion (e.g., within hours or days) so that appropriate PEP recommendations can be made. Countries with IBCM programs have seen a reduction in the amount of unnecessary PEP that is administered and have seen increases in PEP completion rates.^3,8^ In 2012, MSPP, MARNDR, and PRB laid the framework for an IBCM program in just three of Haiti’s 144 communes as a pilot study to determine if IBCM could be integrated into Haiti’s tenuous health systems. The IBCM program first relied on the establishment of a diagnostic laboratory (2012), followed by development of standard operating procedures and case definitions (2012), then training an animal control workforce (2013), and lastly its field implementation (2013).

**Figure 2.**
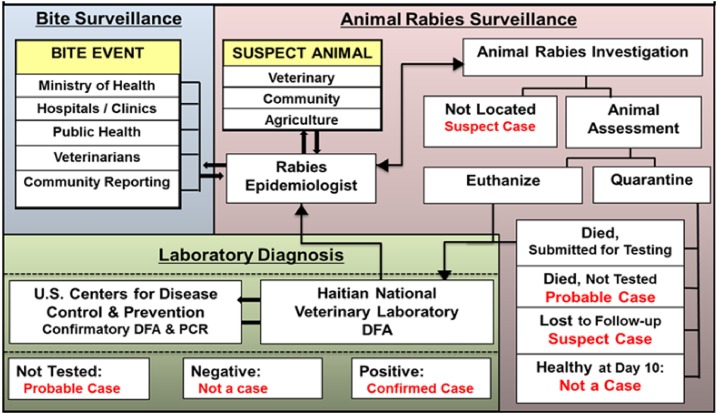
Haiti animal rabies surveillance program.

Animal rabies diagnostic capacity, utilizing internationally recognized techniques, was achieved in mid-2012. Standard operating procedures for an IBCM program were drafted in late 2012. Case definitions for human rabies, bites, and animal rabies were developed (Box 1). Protocols were implemented in early 2013 to link bite events detected through MSPP’s weekly notifiable disease surveillance system to animal rabies investigations led by MARNDR (Figures 1 and 2). The IBCM program utilizes two standardized epidemiologic forms: one for bites seen at health-care facilities (MSPP) and one for the rabies evaluation of the rabies suspected animal (MARNDR) (appendix 1 and 2).

**Box 1:** Case definitions for rabies control program: Haiti

Human rabies	Suspect case: A case compatible with the following clinical description: Hydrophobia, agitation, trembling of the limbs, change in voice, convulsions, hallucinations, fever, or dehydration
	Probable case: A suspected case plus history exposure to a suspected rabid animal
	Confirmed cases: All probable cases confirmed by laboratory
Suspected rabies exposure	Anyone who has been assaulted (bite or scratch) by a species of animal that is known to be able to transmit rabies (dog, cat, bat, mongoose) or an animal showing, signs consistent with rabies (aggression, unprovoked bite, unusual behavior, excessive salivation) at the time of the assault, or within 10 days of the assault
Animal rabies	Suspect case: An animal which has bitten a person or an animal displaying signs consistent with rabies that cannot be further classified based on a risk assessment.
	Dismissed case
	1) Negative rabies diagnosis by direct fluorescent antibody test (or)
	2) Alive and healthy 14 days after the exposure
	Probable case
	1) An animal that bites > 2 people or animals, has a change in behavior, and is dead or unavailable for follow-up 14 days of the exposure (or)
	2) An animal that has shown > 3 signs of rabies and is dead or unavailable for follow-up 14 days of the exposure (or)
	3) An animal that died during rabies quarantine and was not available for testing
	Confirmed case: Positive rabies diagnosis by direct fluorescent antibody test or PCR

PCR = polymerase chain reaction.

**Figure 3.**
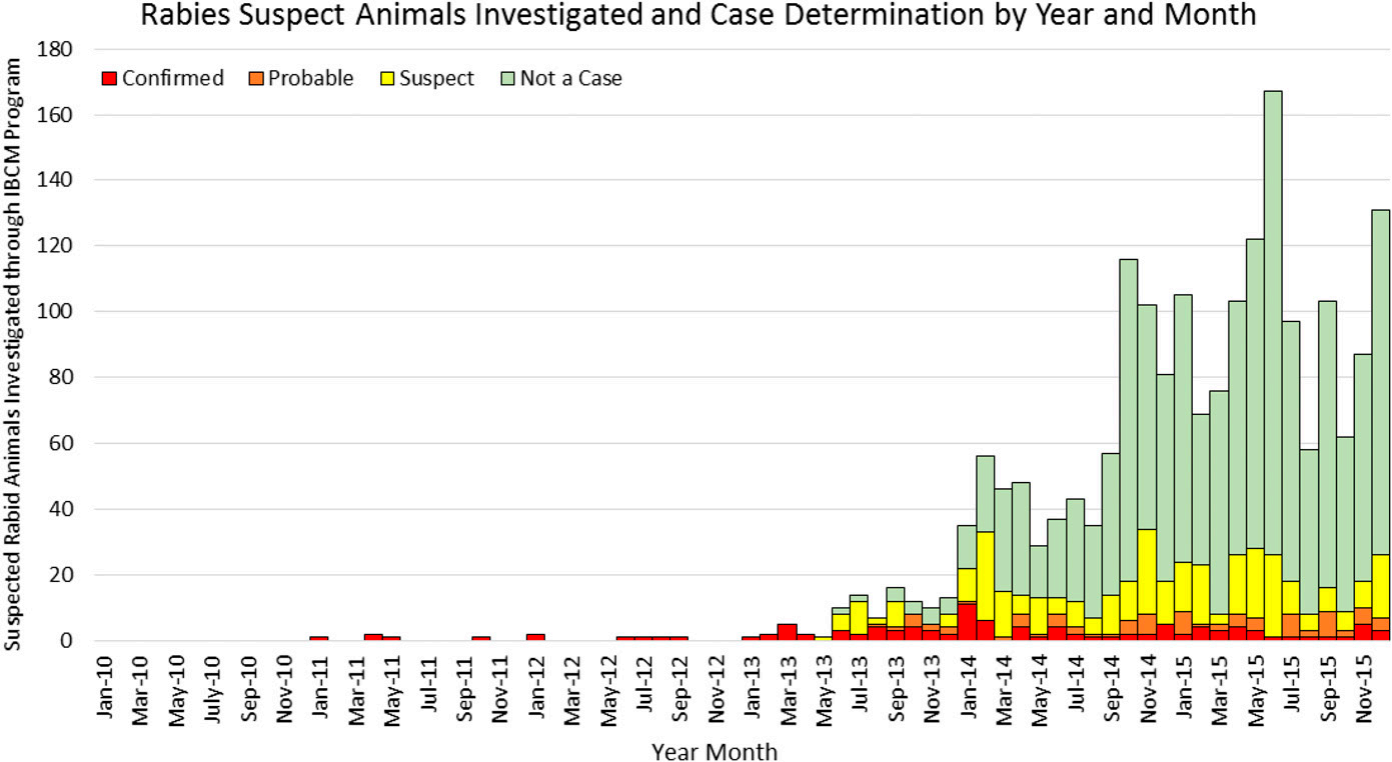
Rabies suspect animals investigated and case determination by year and month, Haiti 2010–2015.

During 2010–2012, only 11 animal rabies investigations were conducted in the entire country (Figure 3). The IBCM pilot program was initiated in January 2013 in one commune in the West Department and was operated by just one animal rabies surveillance officer (ARSO). Within the first 6 months of IBCM operations (January–June 2013), 21 animal rabies investigations were conducted in this one commune (3.5 investigations per month). In July, more than 20 requests for rabies investigation were received. The program was quickly inundated with these bite investigation requests and within 6 months of implementing the IBCM program, PRB and MARNDR added an additional three ARSOs to cover the 20 communes which make up the West Department. From July–December 2013, the team received requests to investigate 72 suspected rabid animals, of which 29 (40.3%) had rabies infection either through diagnostic confirmation (confirmed) or clinical case definition (probable). The rapid success of this program was quickly recognized by MARNDR and MSPP, and with the assistance of CVM, HSI, and PAHO, in 2015 the program was expanded to the departments of Artibonite and Center.

From 2013 to 2015, a total of 1,958 rabies suspect animals were investigated, with 180 determined to have been rabid (9.2%). During this period, the health sector reported 1,324 bite victims to MARNDR for animal investigation. An additional 1,379 bite victims (51%) were reported from entities outside of the health sector or discovered during the course of the field investigations. Of the total 2,703 bite victims served by the IBCM program during the 3-year period, 279 (10.3%) were found to have been exposed to a rabid animal. Only 58 of the 279 (20.8%) people exposed to a rabid animal had received rabies vaccine at the time of the IBCM investigation; all were referred for further medical care as part of this program.

**Figure 4.**
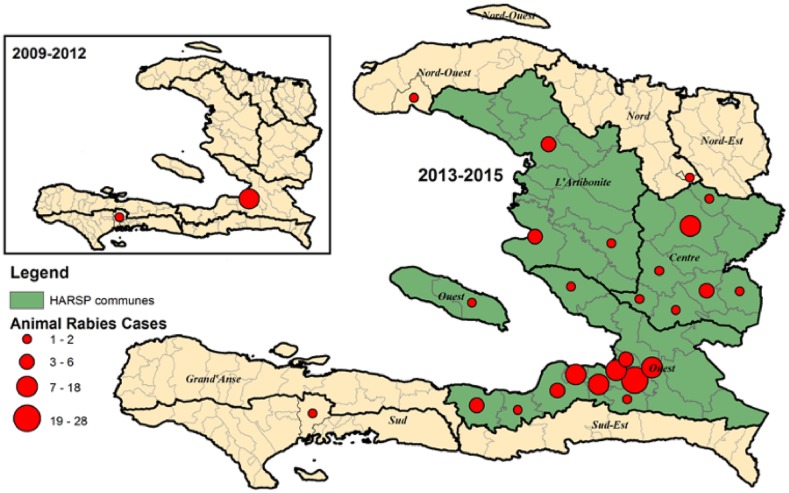
A comparison of rabid animals detected prior to the development of an animal rabies program and after the development, 2009–2012 and 2013–2015.

Since inception of the program in January 2013, Haiti has seen an 178-fold increase in the investigation of suspected rabid animals, a 16-fold increase in detection of rabid animals, a 30% increase in reporting of human rabies exposures, and a 230% increase in PEP adherence among persons who used the IBCM program (data not published).^8^ In the departments where this IBCM program has been implemented, modeled estimates predict a 49% reduction in human rabies deaths based on the improvements in bite detection and health-care-seeking behaviors (data not published). The success of this program was consistent with what was observed after the implementation of a similar program in Bohol, Philippines.^19^ As of April 2016, 45 veterinary professionals have been trained in IBCM, with 14 hired to operate the program in four of Haiti’s 10 departments (68 of 144 communes), covering approximately 72% of Haiti’s population (Figure 4). This IBCM program is currently under consideration for national adoption as part of the 2016–2020 rabies control plan.

## INFORMATION, EDUCATION, AND COMMUNICATION

Preliminary research in Haiti suggests that awareness about rabies infection, prevention, and control is low among the general public, law enforcement, veterinarians, and health-care workers. To increase Haitians’ awareness about rabies prevention, CDC has conducted educational outreach activities in collaboration with MSPP, MARNDR, PAHO, Human Society International, International Fund for Animal Welfare, CVM, Global Alliance for Rabies Control, U.S. Embassy in Haiti, and U.S. military veterinary professionals with the USNS Comfort (a seagoing medical treatment facility that provided medical care and education during a visit to Port-au-Prince in September 2015).

In August 2013, CDC conducted interviews with eight health-care workers in Carrefour and Pétionville to better understand what health-care professionals and general community know about rabies prevention and to get insight into the types of materials, messages, and dissemination methods that could most effectively increase Haitians’ awareness about rabies prevention. The transcripts from these interviews were transcribed, translated, analyzed, and used to identify knowledge gaps and most appropriate and effective messaging tools for the development of education materials for different target audiences. Also, preliminary findings from the interviews supported the presence of knowledge gaps and the need to educate the public and health-care workers about steps they must take to respond quickly and appropriately after someone is bitten by a potentially rabid animal. Education materials were then developed and tested in June 2015, using six focus groups comprised of doctors, nurses, and community leaders from Carrefour and Pétionville. The objectives of the focus groups were to determine if people understood the messages and if the materials would likely motivate them to take action to protect themselves and others from rabies. Findings were supportive of the educational messaging, and to-date CDC and MSPP have developed three posters, two flyers, one hand-out, and a comic book.

In 2015 alone, CDC supported rabies education workshops for more than 200 Haitian doctors, nurses, sanitation officials, and veterinary professionals and worked with partners to develop and disseminate thousands of facts sheets, posters, and comic books that provided clear, culturally appropriate information about rabies to a variety of target audiences: doctors and nurses, veterinary professionals, leadership within MSPP and MARDNR, school teachers, and children aged 6–18. One of these workshops, held in September 2015, used the GARC Rabies Educator Certificate (REC) to train 43 veterinary professionals. For a brief period in late 2015, Haiti had the highest number of REC certified persons in the world. During the workshop, one participant, after learning about rabies signs and symptoms through the REC, alerted workshop trainers to the suspected rabies death of a child in his community. The case was investigated by MARNDR, MSPP, and CDC and resulted in the clinical confirmation of two rabies cases. Eleven other rabies exposed individuals were identified and provided the rabies vaccination series. Rabies prevention billboards, painted murals, and additional communication outreach activities are planned for 2016.

## MASS VACCINATION OF DOGS

The primary means of controlling canine-mediated human rabies deaths is through mass vaccination of dogs, with the goal of attaining at least 70% vaccination coverage to achieve herd immunity. A critical factor in reaching this goal is a broad understanding of the local dog ecology and demography. In 2010, Haiti’s vaccination program operated on the assumption that the country had 400,000–500,000 dogs. Haiti had never conducted a dog census or attempted to describe dog ecology, therefore this estimation was based largely on assumptions. From 2010 to 2012, Haiti procured 250,000 vaccine doses and conducted annual mass vaccination programs (MCVs). Prior to 2013, the MCV included vaccination of cats. However, cats are not a reservoir species for the rabies virus.

In 2012, the World Organization for Animal Health (Office International des Epizooties) and CDC recommended to halt the vaccination of cats during government sponsored MCV to achieve higher vaccination coverage in the dog population. As a result of this policy, in 2013, the vaccination coverage in the dog population (the reservoir for rabies in Haiti) doubled. However, at this time there was still international disagreement as to the true dog population in Haiti and whether doubling the vaccination effort in dogs was enough to reach 70% of the population.

**Figure 5.**
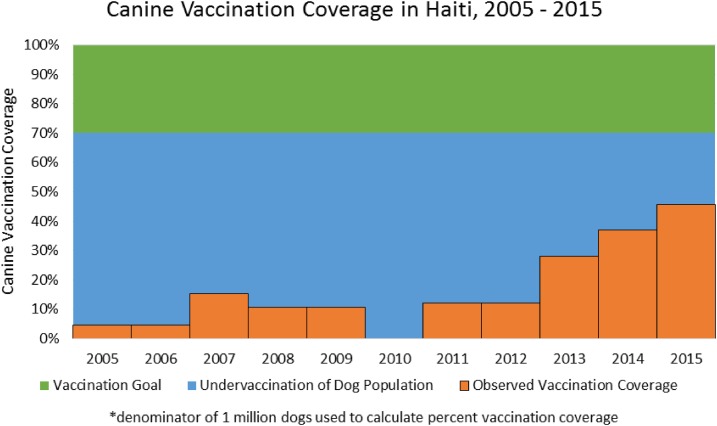
Canine vaccination coverage in Haiti, 2005–2015.

In 2014, CDC, CVM, HSI, and MARNDR conducted a dog population and ecology survey to answer this question. The results (unpublished) indicate that Haiti likely has over 1,000,000 dogs, of which the vast majority are allowed to roam freely, a risk factor for rabies transmission. In 2015, the historical vaccination coverage estimates were updated to reflect this newly recognized dog population (Figure 5). Under the revised dog population estimations, the vaccination coverage from 2010 to 2012 was likely less than 15%. The policy shift to vaccinate only dogs and to procure vaccine based on the higher dog population estimates resulted in an increase in the coverage rate to nearly 50% as of 2015. The goal of the mass vaccination campaign for 2017 is to vaccinate 750,000 dogs. If successful, this would represent the first time that Haiti has reached the target level of 70% and is the first step toward effective rabies control in the country.

## EVALUATING PROGRESS TOWARD RABIES ELIMINATION

In August 2015, the USNS Comfort, CDC, PAHO, and MARNDR coordinated a workshop to evaluate the current status of rabies control in Haiti. For the evaluation, workshop participants used the Stepwise Approach towards Rabies Elimination (SARE) tool developed by the Food and Agriculture Organization and GARC.^21^ This represented the first time that Haiti’s rabies control program was formally evaluated, and the first time since 2011 that the national rabies control program was reviewed by a multidisciplinary group. The SARE tool provides a numerical score (0.0–5.0) to indicate the current status of the program, with 5.0 being a program that has eliminated canine rabies and 0.0 reflecting a program that has no infrastructure to control this disease. The SARE process was used twice, first to reflect the rabies control status in 2010 and second to reflect the status as of August 2015. The SARE score in 2010 was 0 out of 5. In 2015, Haiti’s score had improved to 1.5 out of 5. A subanalysis indicated that a majority of accomplishments were completed through collaborations with MARNDR, CDC, CVM, and PAHO. The results from the 2015 SARE workshop were used to develop a 5-year rabies control strategy (2016–2020).

## CONCLUSION

A country that makes no effort to detect rabies within its borders might, at best, be able to describe the burden through modeled probabilities. Surveillance is necessary to demonstrate the reality of the rabies risk in the country. Inadequate rabies surveillance plagues many canine-rabies endemic countries, and this lack of knowledge about the burden of the disease further contributes to rabies remaining globally neglected. Canine rabies likely became endemic in Haiti shortly after its arrival with French colonists in the late 1600s. It was only after the development of laboratory capacity and an effective surveillance system that the unseen impact of rabies on animals and humans was elucidated, and it is typically the unmasking of this burden that provides the impetus to implement successful control and elimination measures. From 2010 to 2011, Haiti was recovering from a devastating earthquake that disrupted basic health infrastructure and services. Although difficult to verify, rabies likely increased during this period, as canine vaccination efforts stalled and free-roaming dog populations grew. However, control has drastically improved over the past 5 years as a result of strong leadership from MSPP and MARNDR as well as assistance from multiple international organizations. Haiti is now poised to eliminate canine-mediated human rabies deaths in the near future and should serve as a great example to many developing countries struggling with similar barriers and limitations. Continuation of these collaborations will be needed for the foreseeable future to ensure that progress continues, momentum is not lost, and that human rabies of canine origin is eliminated from Haiti by 2030.
